# Significance of Astragaloside IV from the Roots of *Astragalus mongholicus* as an Acetylcholinesterase Inhibitor—From the Computational and Biomimetic Analyses to the In Vitro and In Vivo Studies of Safety

**DOI:** 10.3390/ijms24119152

**Published:** 2023-05-23

**Authors:** Katarzyna Stępnik, Wirginia Kukula-Koch, Wojciech Plazinski, Kinga Gawel, Katarzyna Gaweł-Bęben, Daariimaa Khurelbat, Anna Boguszewska-Czubara

**Affiliations:** 1 Department of Physical Chemistry, Institute of Chemical Sciences, Faculty of Chemistry, Maria Curie–Skłodowska University in Lublin, Pl. M. Curie-Skłodowskiej 3, 20-031 Lublin, Poland; 2 Department of Pharmacognosy with Medicinal Plants Garden, Medical University of Lublin, 1 Chodzki Str., 20-093 Lublin, Poland; virginia.kukula@gmail.com; 3 Department of Biopharmacy, Medical University of Lublin, Chodźki Str. 4a, 20-093 Lublin, Poland; wojtek_plazinski@o2.pl; 4 Jerzy Haber Institute of Catalysis and Surface Chemistry, Polish Academy of Sciences, Niezapominajek Str. 8, 30-239 Kraków, Poland; 5 Department of Experimental and Clinical Pharmacology, Medical University of Lublin, Jaczewskiego Str. 8b, 20-090 Lublin, Poland; kingagawel@umlub.pl; 6 Department of Cosmetology, University of Information Technology and Management in Rzeszów, Sucharskiego 2, 35-225 Rzeszów, Poland; kagawel@wsiz.edu.pl; 7 Department of Pharmaceutical Chemistry and Pharmacognosy, School of Pharmacy, Mongolian National University of Medical Sciences, Zorig Str., Ulaanbaatar 14210, Mongolia; daariimaa@mnums.edu.mn; 8 Department of Medical Chemistry, Medical University of Lublin, Chodźki 4A, 20-093 Lublin, Poland; anna.boguszewska-czubara@umlub.pl

**Keywords:** acetylcholinesterase, molecular docking, IC_50_, free energy, zebrafish, safety, SH-SY5Y, lipophilicity

## Abstract

The main aim of the study was to assess the acetylcholinesterase-inhibitory potential of triterpenoid saponins (astragalosides) found in the roots of *Astragalus mongholicus*. For this purpose, the TLC bioautography method was applied and then the IC_50_ values were calculated for astragalosides II, III and IV (5.9 μM; 4.2 μM, and 4.0 μM, respectively). Moreover, molecular dynamics simulations were carried outto assess the affinity of the tested compounds for POPC and POPG-containing lipid bilayers, which in this case are the models of the blood-brain barrier (BBB). All determined free energy profiles confirmed that astragalosides exhibit great affinity for the lipid bilayer. A good correlation was obtained when comparing the logarithm of n-octanol/water partition coefficient (logPow) lipophilicity descriptor values with the smallest values of free energy of the determined 1D profiles. The affinity for the lipid bilayers changes in the same order as the corresponding logPow values, i.e.,: I > II > III~IV. All compounds exhibit a high and also relatively similar magnitude of binding energies, varying from ca. −55 to −51 kJ/mol. Apositive correlation between the experimentally-determined IC_50_ values and the theoretically-predicted binding energies expressed by the correlation coefficient value equal 0.956 was observed.

## 1. Introduction

The key physiological role of the enzyme acetylcholinesterase (AChE), mainly found at the neuromuscular junctions and cholinergic brain synapses, is the hydrolytic destruction of neurotransmitter acetylcholine (ACh) [1]. The elevated AChE activity affects Ach release due to its rapid hydrolysis to acetic acid and choline. This terminates the impulse transmission at the cholinergic synapses in numerous cholinergic pathways in the central and peripheral nervous systems [2,3]. It is assumed that at high ACh concentrations, each AChE active site hydrolyses 10,000 molecules of substrate per second [1,4]. The AChE inhibitors play an important role in the treatment of neurodegenerative diseases e.g., Alzheimer’s disease (AD) [1].

The cholinergic hypothesis of AD, related to the role of ACh in learning and memorising processes, was developed in the 1970s. The nucleus basalis of Meynert (NBM), identified as the cholinergic centre in the brain, is affected during the pathological aging of people [5]. The neuronal loss within the NBM in the forebrain of AD patients reduces the levels of cortical ACh which leads to cognitive impairments [6]. The cognitive deficit is caused by the dysfunction of the central cholinergic system which in turn, plays a key role in the storage and retrieval of memory in the central nervous system [7,8,9].

Currently, the treatment of neurodegenerative diseases is based on drugs being either AChE inhibitors or antagonists of N-methyl-D-aspartate (NMDA) receptors [10]. Unfortunately, none of the current therapeutic strategies can successfully stop the progression of the disease in the early stages. Therefore, the main task of available drugs is to alleviate the symptoms of the disease and slow it down. These drugs include, among others, galantamine [11,12], rivastigmine [13,14] donepezil [15] and memantine [16]. Frequent drug resistance as well as undesirable effects of their long-term use and insufficient effectiveness during long-term therapy have resulted in the need to search for new drug candidates, including AChE inhibitors, mainly of plant origin. Although there are some registered AChE inhibitors already on the market, there is still a need for the identification of novel drug candidates for the same applications. Currently-used medicines cause significant side effects. Dizziness, nausea, cognitive and digestive system disorders and most important lytolerance to the used dosageare only some of the drawbacks of the existing therapeutic strategies [17].

The richness of traditional Chinese medicine can be a source of searching for new AChE inhibitors e.g., *Huperzia serrata*, whose active compounds, named *Lycopodium* alkaloids, have a wide spectrum of medicinal applications. Huperzine A is considered to be the most potent, reversible and selective AChE inhibitor which can make it useful in the treatment of dementia and other relevant cognitive impairments [10,18,19]. Galantamine, which is an active ingredient of many plants from the *Amaryllidaceae* family, has a similar AChE-inhibitory effect. This substance is approved by the Food and Drug Administration in the United States as well as by the State Food and Drug Administration in China for the treatment of mild to moderate AD [20]. It was also reported that tenuifolin from *Polygala tenuifolia* can enhance cholinergic neurotransmission as well as inhibit AChE activity [21].

In this research, triterpenoid saponins—the active compounds contained in the *Astragalus mongholicus* roots—have been tested and evaluated for AChE-inhibitory potential. *Astragalus*, an annual flowering plant of *Fabaceae*, has been widely used by humans for cardiovascular inflammation, digestive and renal abnormalities as well as fatigue and weakness since the most ancient periods of development of medical science. Over 2000 species of *Astragalus* are spread throughout the world, while approximately 70 species are registered in Mongolia alone [22].

In our previous study [23], the ability to cross the blood-brain barrier (BBB) of triterpenoid saponins derived from the *Astragalus mongholicus* roots was investigated. It was noted that the most active compound among those tested is astragaloside IV (A IV). In post-mortem research, carried out on the brain tissues of mice, the ability of astragaloside IV to cross the BBB was confirmed and thus the logBB value was determined (0.49).

Keeping in mind the results obtained in our previous study, here we aimedat carrying on the research into the significance of astragalosides as AChE inhibitors. To do so, a TLC bioautography assay, molecular dynamics simulations and molecular docking as well as biomimetic research were carried out to show the greatest potential of A IV to inhibit AChE. The studies were complemented with the assessment of A IV safetyin vitro using the SH-SY5Y human neuroblastoma cell line as well as in vivo using the zebrafish model [24].

## 2. Results

### 2.1. Biomimetic Models for Lipophilicity Determination

To determine lipophilicity of the tested astragalosides, the biomimetic-High Performance Liquid Chromatography (HPLC) systems with a cholesterol-bound (CHOL) stationary phase as well as the Immobilized Artificial Membrane (IAM) column were used. These stationary phases have pseudomembrane properties and therefore are widely applied in chromatographic practice to determine lipophilicity of various groups of compounds, including newly synthesized ones [25,26,27,28,29].

Based on the Soczewiński–Wachtmeister Equation (1) [30], the logarithms of retention factors extrapolated to pure water (logkw) values were calculated. These values can be considered as an alternative to the n-octanol/water partition coefficient recognized as the lipophilicity descriptor [31].

logk = logkw − sφ(1)

where logk is the logarithm of the retention factor in the mixed effluent systems; φ is the volume fraction of organic modifier in the mobile phase; s is the slope characteristic of a given solute in the chromatographic system. The logkw, s, and R^2^ values obtained from Equation (1) for the tested chromatographic systems are presented in Table 1.

### 2.2. HPLC-MS Fingerprinting of Extracts

**Figure 1:**
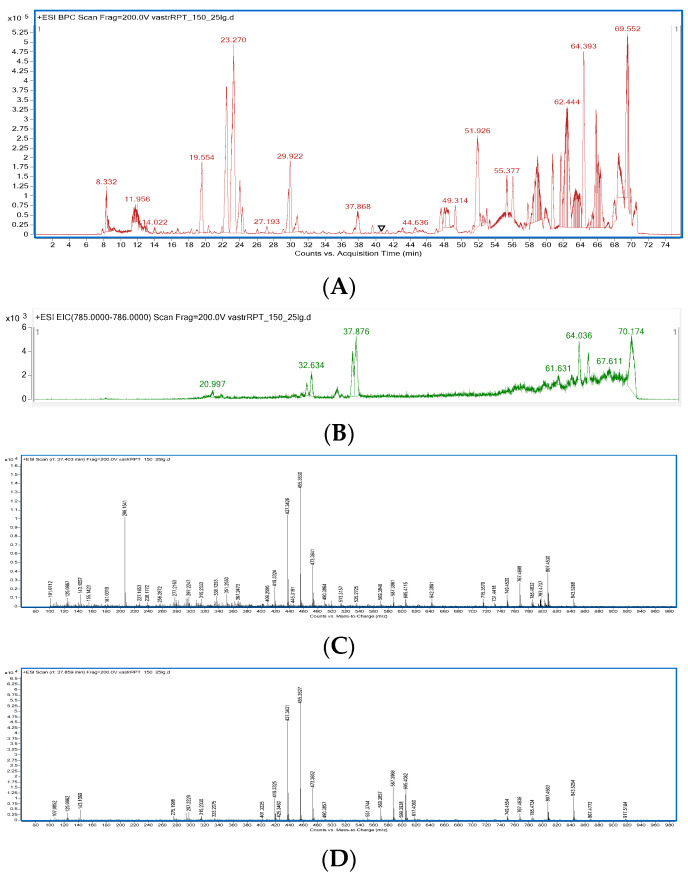
Total ion chromatogram of 50% methanolic extract from the roots of *Astragalus mongholicus* in the positive ionisation mode (**A**), extracted ion chromatogram of astragaloside III and IV (*m/z* of 785) (**B**), mass spectrum of astragaloside IV (**C**), mass spectrum of astragaloside III (**D**).

The obtained 50% ethanolic extract from *Astragalus mongholicus* roots was analysed for its composition by the HPLC-MS instrument to determine the presence of astragalosides III and IV, which were found to exhibit inhibitory properties in the AChE assay. The fingerprint of the analysed extract together with the fragmentation pattern of both compounds are presented below in Figure 1.

### 2.3. Molecular Dynamics

**Figure 2:**
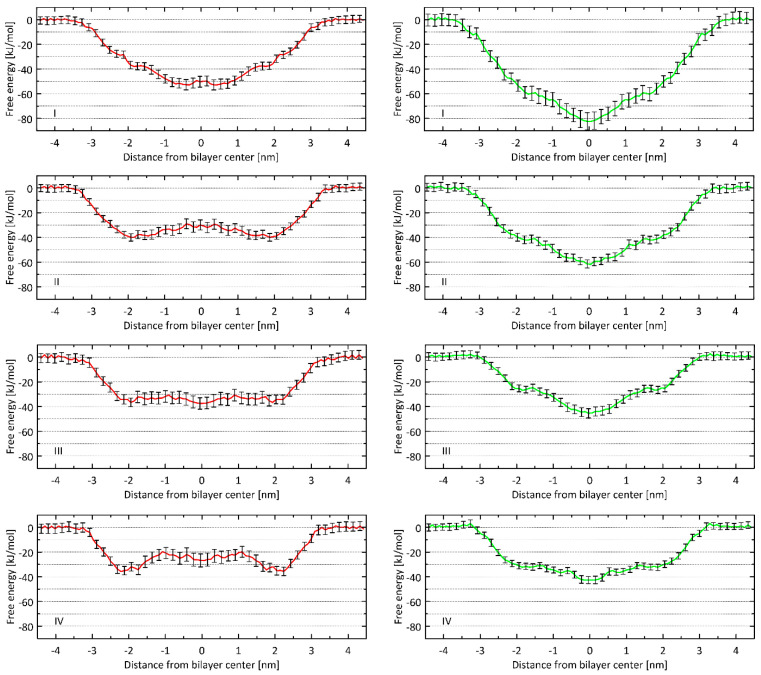
1D free energy profiles associated with the permeability of astragalosides I–IV through the lipid bilayer. Two types of homogeneous bilayers were considered: composed either of POPC (red lines, l-h-s panels) or POPG (green lines, r-h-s panels). The error values (vertical bars) were estimated by the bootstrapping method.

Molecular dynamics simulations were performed to assess the affinity of the tested compounds for POPC and POPG-containing lipid bilayers, which in this case are the models of the blood-brain barrier. Taking into account the permeability of astragalosides I–IV through the biological membranes, free energy profiles through the lipid bilayer were determined (Figure 2).

### 2.4. TLC Bioautography

TLC bioautography was used to determine the biological properties of the major constituents of *Astragalus mongholicus* roots. In the assay on the inhibitory properties towards the acetylcholinesterase enzyme, astragalosides were found to be active.

**Figure 3:**
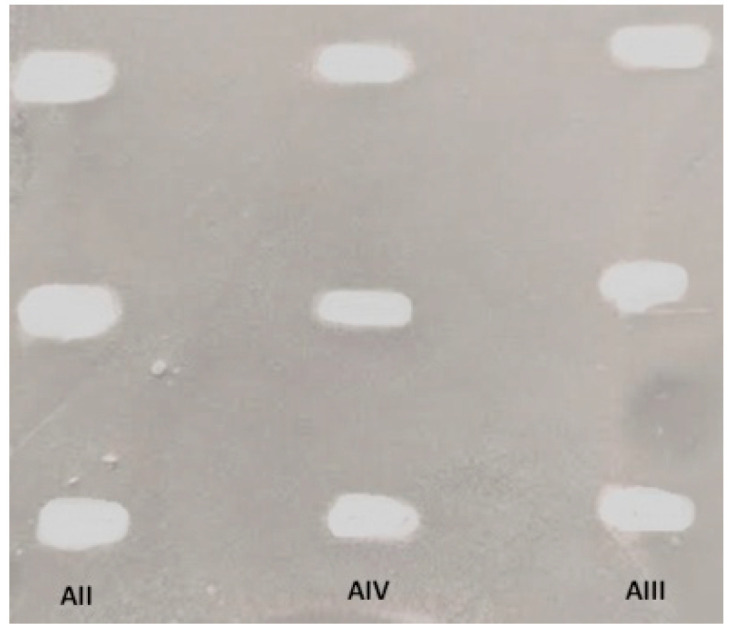
Results of the TLC-bioautography assay for the inhibition of acetylcholinesterase enzyme in daylight on the silica-gel-covered TLC normal phase. The TLC plate shows different concentrations of the tested astragalosides: II (A II), III (A III), and IV (A IV).

In this study, the reference compounds of astragalosides II (A II), III (A III) and IV (A IV) at different concentrations were injected into the normal phase TLC plate covered with silica gel. Each experiment was repeated three times and the average values of the obtained peak areas corresponding to different injections of pure astragalosides were taken for the calculation of IC_50_ values of the saponins. For the three astragalosides: II, III and IV the following calibration curve equations were obtained: y = 70,773,628.9063x − 62,644.2995 (R^2^ = 0.9732), y = 33,106,972.8125x − 67,429.4097 (R^2^ = 0.9771), and y = 48,308,195.3125x + 3538.3021 (R^2^ = 0.9981), respectively. Finally, the obtained IC_50_ values were: 0.00488 mg for astragaloside II, 0.00330 for astragaloside III, and 0.00316 for astragaloside IV. The respective chromatogram is presented in Figure 3.

The results of the TLC bioautography assay encouraged the authors to focus on the analysis of molecular aspects related to the BBB permeation of astragaloside IV and on the character of its relations with the AChE enzyme. For the compounds A II, A III, and A IV the IC_50_ values were obtained experimentally using the TLC bioautography assay (5.9 μM; 4.2 μM, and 4.0 μM, respectively).

### 2.5. Molecular Docking

**Figure 4:**
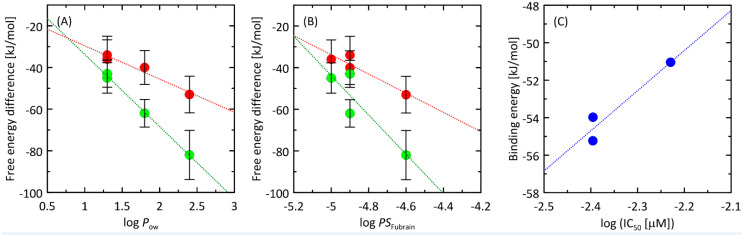
(**A**) Correlation between the logP_ow_ values (taken from [32]) and the smallest value of the free energy profiles associated with the permeability of the studied compounds through the lipid bilayer. The lipid bilayer was composed of either POPC molecules (red points) or POPG molecules (green points). The error bars correspond to the combined bootstrapping errors determined for the smallest value of the energy and the value for the molecule located outside the bilayer. (**B**) The same as in (**A**) but the correlation calculated for the logPS_Fubrain_ values. (**C**) Correlation between experimentally-determined IC_50_ values (recalculated as log(IC_50_)) and the theoretical ligand-AChE binding energies.

**Figure 5:**
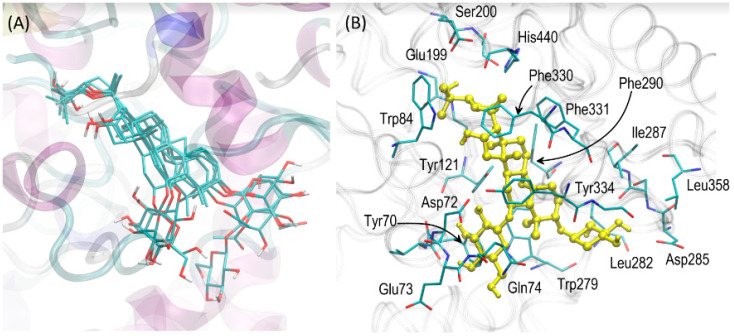
(**A**) Superposition of the most favourable poses of all ligands interacting with AChE. (**B**) The most favourable location of the astragaloside IV molecule bound to AChE. The ligand molecule is shown in ball-and-stick representation whereas all the closest amino-acid residues (of the distance not larger than 0.38 nm) are represented by thin sticks. The description of the interaction types is given in the text. The residue numbering is compatible with the PDB:3EVE record.

All compounds exhibited high and also relatively similar magnitudes of binding energies, varying from ca. −55 to −51 kJ/mol (Figure 4A,B). The energy values were negative which confirms the strongly favourable binding in all considered cases. There was also a positive correlation observed between the experimentally-determined IC_50_ values (recalculated as log(IC_50_)) and the theoretically-predicted binding energies (Figure 4C) expressed by the correlation coefficient value equal to 0.956. However, due to the small number of data points and also the small scatter across the sets of both the experimental IC_50_ and theoretical energies, such correlation can be coincidental. Nevertheless, the docking results are in qualitative agreement with the experimental results, confirming the large potency of astragalosides to be bound into the active site of AChE (Figure 5).

### 2.6. In Vitro and In Vivo Safety Studies

**Figure 6:**
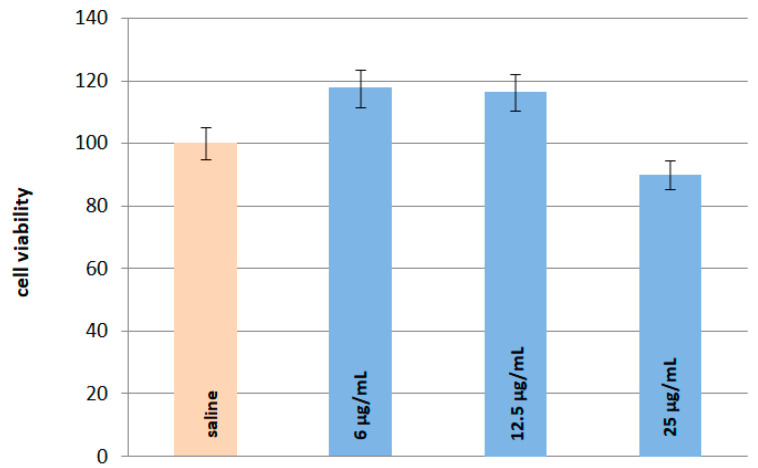
Effect of astragaloside IV (6, 12.5, and 25 μg/mL) on viability of SY-SY5Y human neuroblastoma cells; the histogram shows the mean cellular viability ± SD in comparison with the control saline-treated cells.

To evaluate in vitro safety of the most potent AChE inhibitor among the tested saponins (A IV) the SY-SY5Y human neuroblastoma cell line was used (Figure 6). This cell line is as a well-known model of dopaminergic neurons in Parkinson’s disease [33].

**Figure 7:**
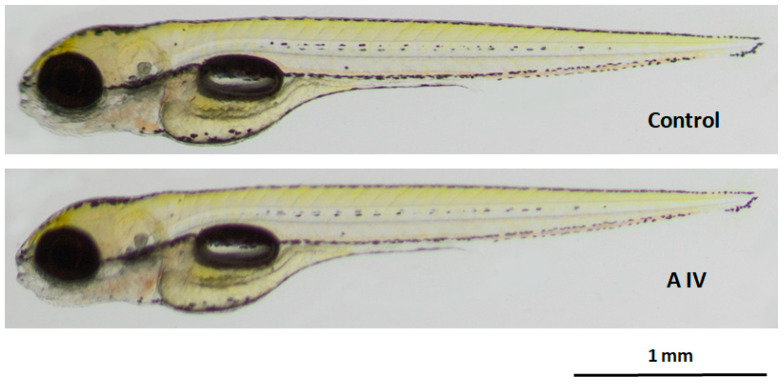
Representative photo of control and astragaloside IV-treated (25 µg/mL) larvae after the 95 h long incubation. The scale bar 1 mm.

Since the zebrafish (*Danio rerio*) is the embryonic and larval model for screening safety as well as potential toxicity [34] of a compound of interest, herein it was used to assess the toxicity of astragaloside IV in vivo. For this purpose, zebrafish embryos were incubated in 25 µg/mL of astragaloside IV from 1 up to 96 hpf (Figure 7).

## 3. Discussion

The previous studies showed that the metabolites of *Astragalus mongholicus* Bunge are able to cross the BBB [23], and are characterized by a small toxicity [35]. In the doses of 50, 100, and 200 mg/kg the plant did not interfere with the locomotor activity or situational anxiety as measured in the elevated plus maze test. On the other hand, in these doses *Astragalus mongholicus* Bunge induced the pharmacological actions that included a significant suppression of the number and duration of seizures in the pentylenetetrazole (PTZ)-induced model of epilepsy and in the subchronic application it showed an impact on the number of neurons in the CA1 (decrease) and CA3 area (no impact) of the hippocampus [35].

The above-mentioned information encouraged the authors to study the potential of astragalosides—the major secondary metabolites present in the plant—concerning their potential application as an AChE inhibitor. The previous studies [23] showed a considerable potential of astragalosides in the inhibition of acetylcholinesterase enzyme. The aim was to evaluate the ability of astragalosides to cross the BBB as well as to compare which of the astragalosides exhibited greater inhibition against AChE based on the peak area values (the IC_50_ values were not shown). That is why the results presented herein are the continuation of the research on their potential mechanism of action, molecular grounds for their AChE inhibition activity, strength in action and finally, toxicity.

As the previous study [23] proved the highest biological potential of astragalosides III and IV compared with the other tested saponins. In this research their presence was confirmed in the whole extract obtained from the 50% ethanolic one from the root of *Astragalus mongholicus* by the HPLC-ESI-QTOF-MS/MS instrument. Both compounds were visualized as the major metabolites clearly visible in the middle part of a mass chromatogram similarly to the studies by other authors [36,37].

The bioactivity of astragaloside IV towards diseases of the central nervous system has been studied by other authors previously. In terms of its potential to favour the treatment of cognitive impairment, anti-inflammatory action and the attenuation of oxidative stress seem to be the most important and so far proven actions of this saponin. In the studies by Zhang and co-investigators [38], astragaloside IV was confirmed to improve the condition of neuronal damage by the regulation of the Nrf2/Keap/HO-1/NQO1 pathway. According to Meng et al. [39], the saponin showed a protective effect towards the neurons treated with hypoxia reducing the calpain-1 and HIF-1a expression and affecting the downregulation of the calpain-1/HIF-1α/caspase-3 pathway. In addition tothe other reports on its influence on mitochondrial functions, the modulation of PI3K/Akt [40], AGEs/RAGE/NF-κB Axis [41], and PARγ/BDNF signalling pathways, these findings contribute to the creation of an interesting and comprehensive bioactivity profile of the compound.

### 3.1. Biomimetic Models for Lipophilicity Determination

Strong linear relationships between the logk and logkw values were obtained for all the tested compounds in all the chromatographic systems with the average R^2^ value 0.980 and 0.983 for the IAM and CHOL systems, respectively. In addition, these relationships also confirmed the congeneric nature of the investigated compounds. Moreover, the previously in silico calculated logPow values are as follows: 5.020, 4.459, 3.767, 3.757 for A I, A II, A III, and A IV, respectively [23]. Comparing the biomimetic-chromatographic lipophilicity descriptors (logkw) values with the computational ones (logPow), it can be concluded that the smallest differences between them were observed in the system with the cholesterol-bound stationary phase (CHOL). There are the most convergent relationships between the lipophilicity of the tested saponins determined in silico and using the IAM stationary phase. The most lipophilic compound is A I whereas A IV is the least; however, the difference between these values is 0.986. Moreover, it was observed that more hydrophobic molecules reveal greater s values which is in line with the background retention theory in which the s values are related to the solute/mobile phase and the solvent/stationary phase net interactions [31]. Both the IAM and CHOL systems determined the lipophilicity of the studied astragalosides to be similar to the computational method.

### 3.2. HPLC-MS Fingerprinting of Extracts

Both compounds were present in the centre of the chromatogram, around the 37th minute with astragaloside IV eluted first (37.4 min) and astragaloside III eluted second (37.8 min). The sequence of elution of these two saponins was estimated based on the comparison with the standard of astragaloside III, and also based on the previously published data of Zu et al. [42].

Moreover, both compounds identified in the total extract were characterized by a similar fragmentation pattern as described by the above-mentioned authors. The characteristic signals in their mass spectra were as follows: 785 u—as the precursor ion [M+H]^+^, 605 u—as the molecular ion with glucose detached from the structure [M+H-Glu]^+^, 473—the molecular ion with glucose and xylose detached from the molecular ion [M+H-Glu-Xyl]^+^, 455—the molecular ion with removed glucose, xylose and water [M+H-Glu-Xyl-H_2_O]^+^, 437—the molecular ion with no glucose, xylose and two water moieties [M+H-Glu-Xyl-2 H_2_O]^+^, and 419—the molecular ion with removed glucose, xylose and three groups of water [M+H-Glu-Xyl-3H_2_O]^+^. Both compounds had also a characteristic ion at 143 that was due to the presence of the substituent containing a substituted oxolan group.

### 3.3. Molecular Dynamics Simulations

Molecular dynamics simulations were performed to determine the free energy profiles associated with the permeability of astragalosides I–IV through the lipid bilayer. All determined free energy profiles (Figure 2) confirm that astragalosides exhibit great affinity for the lipid bilayer. This effect is expected to be due to the positive values of the logPow parameter. Moreover, a good correlation is obtained when comparing the logPow values with the lowest value of free energy on the determined 1D profiles (Figure 4A). Thus, the affinity for the lipid bilayers changes in the same order as the corresponding logPow values, i.e.: I > II > III~IV. The same trend is characteristic of both POPC- and POPG-containing lipid bilayers, although the affinity of astragalosides for the bilayer is notably larger in the latter case. In none of the cases was the free energy barrier associated with the immersion into the bilayer observed. Instead, only in the case of astragalosides II, III and IV and the POPC membrane did small (II, III) or moderate (IV) barriers appear, located either at or around the centre of the bilayer.

The results of molecular dynamics suggest that all the studied compounds have a tendency to accumulate in the lipid bilayers and the permeability rate is associated with leaving the bilayer rather than entering it. The minimal energies determined from free energy profiles also correlate reasonably well with the brain/plasma equilibration rate expressed as the logPS_Fubrain_ parameter (Figure 4B) [23].

### 3.4. Molecular Docking

The results of the docking study were also analysed with respect to the mechanistic interaction pattern, significant in the context of interpretation of the obtained binding energies and the mechanism of inhibition. The summary given below consists of analysing the ligand-protein contacts that take place if the distance between any corresponding atom pair is smaller than the arbitrarily accepted value of 0.38 nm. Figure 5A shows the superposition of all the most favourable ligand poses whereas Figure 5B shows the most essential residues involved in the ligand-protein interactions. The extremely close match between the superposed structures allows the transfer of most of the conclusions drawn on the basis of the most potent compound (i.e., astragaloside IV) to the remaining ones.

The detailed pattern of the ligand-enzyme interactions is illustrated in Figure 5B, with the exemplary A IV displaying the smallest IC_50_ value. All ligands prefer roughly the same binding position in the enzyme cavity which enables them to block the catalytic site (the proximity of the ligand to the two catalytic residues, His440 and Ser200, can be observed). Moreover, blocking is achieved by limiting the access to the catalytic site as the ligand molecule is located in the vestibule of the catalytic triad. The central fragment of the ligand molecule (composed of aliphatic, cyclic moieties) interacts with the aromatic cluster of sidechains, created by Phe290, Phe330, Phe331, Tyr121, and Trp279. These contacts have a character of the CH-π interactions. The two sugar moieties (glucose and xylose, both in the pyranose form) interact with more polar residues, located closer to the binding cavity entrance (e.g., Asp285, Glu73, Asp72 or backbone fragments of Gln74 and Leu282). Some of these contacts occur via the hydrogen binding. Interestingly, the glucose residue interacts with the two aromatic sidechains of Tyr70 and Trp279, exhibiting the CH-π interactions characteristic of non-charged carbohydrate-protein binding [43]. The close presence of the non-polar residues of Ile287 and Leu358 seems to be an opportunistic consequence of previously-listed, more intensive interactions. Due to the location of the carbohydrate residue with respect to the binding cavity (they are likely to be solvent-exposed) and the inherent conformational flexibility of the glycosidic bonds, it can be speculated that the carbohydrate-protein interactions may be not as relevant for the overall binding strength in comparison to the interactions involving the central part of the ligand molecule. This is indirectly confirmed by the extremely similar values of both binding energy and IC_50_ obtained for all the considered compounds, in spite of the fact that the largest differences of chemical and structural character between particular astragalosides involve regions of substitution by carbohydrate moieties. Finally, the opposite part of the ligand molecule (containing the substituted oxolane moiety) interacts with the sidechains Trp84, Phe330, Glu199, Ser200, and His440; the two latter residues belong to the catalytic triad. The character of interactions varies between hydrogen binding (Glu199 and Ser200) and CH-π interactions (His440, Trp84, Phe330). These contacts are well-conserved among the set of considered molecules, as indicated by the small scatter of the corresponding moieties on the superposed structures of the docked ligands (Figure 5A).

### 3.5. In Vitro and In Vivo Safety Studies

After administration of astragaloside IV at doses of 6 and 12.5 μg/mL cell viability is slightly higher than the value for the control group (saline), while at the highest dose used (25 μg/mL) it is slightly lower. This means that no significant decrease in the cellular SH-SY5Y viability was observed for all analysed experimental conditions (Figure 6) which is consistent with the previous findings showing a lack of A IV cytotoxicity in vitro [44].

In the case of in vivo studies on the zebrafish embryos 1, 2, 3 and 4 days post-fertilization (dpf), there was no difference between the control and experimental groups in the mortality rate (*p* > 0.05). In addition, astragaloside IV (25 µg/mL) did not affect the larval hatchability of 3- and 4-day-old fish (*p* > 0.05). After the 95 h long exposition to astragaloside IV, there was no difference between the tested groups in relation to the scored morphological abnormalities (*p* < 0.05). The fish looked identical to the control counterparts (Figure 7). Also, in the astragaloside IV-exposed fish the touch-evoked response was normal. Thus, one may conclude that 25 µg/mL of astragaloside IV is safe for developing zebrafish—it does not affect hatchability, morphology and muscle function as well as performance.

## 4. Materials and Methods

### 4.1. The Analytes

The chemical structures of the investigated astragalosides I–IV (A I–A IV) from the *Astragalus mongholicus* roots are presented in Table 2.

### 4.2. Plant Material and Determination of Astragalosides

The roots of *Astragalus mongholicus* were obtained from Ulaanbaatar (Bayangol district) in July 2017. They were authenticated by Dr. Otgonbataar Urjin from the Mongolian National University of Medical Sciences. Dried and ground roots were extracted by a mixture of water–methanol (50:50 *v/v*) as a result of overnight maceration according to the protocol described in our previous study [23]. The extract was evaporated to dryness at 45 °C using a rotary evaporator. Later the dried residue was used for the identification of saponins according to the protocol described in the European Pharmacopea Edition 8.0. The obtained residue was re-dissolved in water and shaken with butanol 4times. The joined butanol fractions were shaken vigorously twice with a 15% solution of ammonia, to enable the formation of astragaloside IV [45]. Later the butanol fractions were joined, evaporated to dryness, resuspended in methanol and subjected to HPLC-MS analysis after being filtered through nylon syringe filters (0.2 µm pore diameter).

### 4.3. Chemicals

All compounds used in this research were >95% pure by HPLC analysis. Astragaloside IV from the aqueous methanolic extract of *Astragalus mongholicus* roots was isolated according to the procedure described in our previous study [23]. The pharmacopoeial standards of astragalosides I-III were purchased from Sigma Aldrich (Sigma Aldrich, St. Louis, MO, USA; p.a.). All the chromatographic measurements were performed using acetonitrile (ACN; Sigma Aldrich, St. Louis, MO, USA; p.a)–phosphate buffer at pH 7.4. The buffer components i.e., citric acid and disodium hydrogen phosphate (Na_2_HPO_4_) were purchased from Sigma Aldrich (Sigma Aldrich, St. Louis, MO, USA; p.a.). Distilled water was obtained from the Direct-Q3 UV apparatus (Millipore, Warsaw, Poland).

### 4.4. Chromatographic-Biomimetic Equipment and Conditions

The Shimadzu Vp liquid chromatographic system (Shimadzu, Kyoto, Japan) equipped with an LC 10AT pump, an SPD 10A UV-Vis detector, an SCL 10A system controller, a CTO-10 AS chromatographic oven and a Rheodyne injector valve with a 20 µL loop was applied in the HPLC measurements.

The solutions of the isolated A IV as well as pharmacopoeial standards of A I–A III were prepared in methanol (Merck, Darmstadt, Germany; p.a.) at a concentration of 1 mg/mL. All the astragalosides proved to be in the neutral form in the solution under experimental conditions. The optimization process of the chromatographic separation was made before the experiment. The flow rate of the mobile phases was established to be 1 mL/min and the temperature was set at 20 °C. The tested compounds were detected with UV light at λ = 203 nm.

The IAM.PC.DD2 column (IAM; 100 × 4.6 mm i.d., 10 µm; Regis Technologies, Morton Grove, IL, USA) and cholesterol-bonded (CHOL; Cosmosil; 75 × 2 mm i.d., 2.5 µm; Genore, Warsaw, Poland) were used as the stationary phase while the buffered solutions of acetonitrile (ACN) were used as mobile phases. In each measurement the composition of the mobile phases was: 0.3; 0.4; 0.5; 0.6 *v/v* ACN-buffer. The buffer was prepared from solutions of both Na_2_HPO_4_ (0.02 mol/dm^3^) and citric acid (0.01 mol/dm^3^).

The dead-time values were measured from the citric acid peaks. All the reported logarithms of the retention factors were measured three times. The values of the peak asymmetry factor were in the acceptable range.

### 4.5. HPLC-MS Based Identification of Astragalosides I–IV in Astragalus mongholicus

The fingerprinting of the *Astragalus mongholicus* 50% methanolic extract was performed using a platform (G320AA) composed of the HPLC chromatograph 1200 Series (Agilent Technologies, Santa Clara, CA, USA) containing a binary pump, a degasser, an autosampler and the PDA detector as well as the TOF-MS mass detector (6210 MSD TOF) with the ESI dual spray ionization source. For the separation the Zorbax Stable Bond C-18 chromatographic column (150 × 2.1 mm, dp = 3.5 µm; Agilent Technologies) was operated in the following gradient mode of acetonitrile with 0.1% of formic acid (solvent B) in 0.1% aqueous solution of formic acid (solvent A): 0 min, 1% of B in A; 10 min, 45% of B in A; 60 min, 90% of B in A; 61 min, 1% of B in A; 75 min, 1% of B in A. The post run was set at 10 min., the thermostat temperature at 25 °C, the monitored wavelengths at 203, 210, 254, 280 and 365 nm and the flow rate at 0.2 mL/min. The following settings of the mass spectrometer were used: fragmentation energy: 200V, gas temperature: 350 °C, gas flow: 10 L/min, nebulizer pressure: 30 psi, capillary voltage: 4000 V, m/z range: 100–1000 u. The Mass Hunter Workstation program (version B.10.00, Agilent Technologies) was used to record and handle the data.

### 4.6. Molecular Dynamics

The molecular dynamics (MD) simulations were carried out using the GROMOS 53a6 force field [46] and within the GROMACS 2016.1 package [47]. The simulated systems included one astragaloside (I-IV) molecule and lipid bilayer, immersed in the simulation box containing water molecules (SPC model [48]) and neutralized by the appropriate number of sodium ions when necessary. Two types of lipid bilayer were considered: either the POPC or POPG bilayer. The parameters for phospholipids were adopted from ref. [49] whereas those for astragalosides were generated by the Automated Topology Builder online server [50]. The simulations were made under periodic boundary conditions based on the rectangular computational boxes of initial dimensions equal to ca. 8 × 8 × 12 nm. After the geometry optimization and equilibration, the non-equilibrium pulling simulation was initiated, aiming at forced migration of the astragaloside molecule from the bulk solution, through the lipid bilayer to the bulk solution again (force constants of the associated harmonic potential were equal to 5000 kJ mol^−1^ nm^−2^ whereas the pull rate was 0.01 nm ps^−1^). The position of the astragaloside molecule along the Z axis of the box (perpendicular to the bilayer) was accepted as the coordinate. Along the reaction coordinate, 40 windows were selected in the range of ca −4.5–4.5 nm (where the centre of the bilayer corresponds to the zero value) and 40 independent simulations were initiated with the umbrella harmonic potential fixed at the distance between the centre-of-mass of the astragaloside molecule and the centre of the box; the accompanying force constant was equal to 5000 kJ mol^−1^ nm^−2^. The data within each window were collected every 2 ps during 40 ns. After removing the first 5 ns for equilibration, the 1D free energy profiles were constructed with the weighed histogram analysis method (WHAM) [51] as implemented in GROMACS (gmx wham) [52]. The statistical uncertainties of the energy profiles were estimated using the Bayesian bootstrapping of complete histograms [52]. The equations of motion were integrated with the time step of 2 fs. The P-LINCS algorithm was applied to constrain the lengths of all bonds [53]. The temperature was maintained close to its reference value (310 K) by applying the V-rescale thermostat [54] whereas for the constant pressure (1 atm, semiisotropic coordinate scaling) the Parrinello–Rahman barostat was used with a relaxation time of 1 ps [55]. The centre of mass motion was removed at every step. Electrostatics were treated with the particle-mesh Ewald (PME) [56], using the short- range cut-off of 1.2 nm, and the van der Waals interactions were switched off between 1.0 and 1.2 nm.

### 4.7. Determination of the IC_50_ Values of Astragalosides

The TLC-bioautography method was used to determine the IC_50_ values of the tested saponins. Owing to the application of this technique, bright spots of active compounds were registered against the dark background. The TLC chromatogram was obtained on aluminium-covered normal phase TLC plates (Merck, Darmstadt, Germany, silica gel 60 F_254_).

To calculate the strength of the astragalosides, the IC_50_ values were determined for astragalosides II, III, and IV. For this purpose, 1 mg/mL solutions of all pure compounds were sprayed on the TLC plate using the Camag Linomat 5 (Muttenz, Switzerland) with the decreasing volumes in the range of 0.002–0.008 mg of compounds per spotevery 1 cm.

Later the TLC plate was subjected to the TLC-bioautography assay towards inhibitory properties against the AChE enzymeas described previously [23]. Briefly, first the TLC plates were sprayed with asolution of 30 mg/20 mL of 1-naphtyl acetate. After drying the TLC plate under the laboratory hood in the air, the TLC plate was sprayed with a solution of AChE enzyme (3 U/mL) that was diluted in the Tris buffer (pH = 7.8) with the addition of bovine serum (500 mg/500 mL of water). The TLC plate was incubated for 10 min at 37 °C in the humidified laboratory drier. Then the TLC plate was removed from the heater and left in the air to dry. Subsequently, the silica gel plate was sprayed with asolution of Fast Blue B salt (1.25 mg/mL)–a derivatiser–which resulted in the colouration of the background. The chromatogram was analysed by the WinCats (v. 1.4, Camag) program and the peak areas of every spot were determined. Based on the numerical values obtained corresponding to the intensity of discoloured zones measured with the peak areas, the calibration curves were determined and the IC_50_ values were calculated as half of the peak area from the biggest spot that stayedwithin the linearity range area.

### 4.8. Molecular Docking

The astragaloside I–IV molecules were created using the online SMILES translator [57] and subsequently optimised within the UFF force field [58] (5000 steps, the steepest descent algorithm) and the Avogadro 1.1.1 [59] software. The flexible and optimised ligand molecules were docked into the binding pocket of the protein structure found in the PDB database (PDB:1EVE). Docking simulations were performed in the AutoDock Vina software (AutoDock Vina 1.1.2; vina.scripps.edu). [60]. The procedure was performed within the cuboid region of the dimensions 22 × 30 × 24 Å^3^ which covers the co-crystallized ligand present in the considered PDB record as well as the closest amino-acid residues that exhibit a contact with this ligand. All the default procedures and algorithms implemented in AutoDock Vina were applied during the docking procedure. The rotatable torsional angles in both ligand molecules and the selected amino-acid sidechains within the binding cavity (Tyr334, Phe330, Phe75, Trp84, Glu199, Ser200, Tyr70, Tyr121, Trp279, Phe290, Phe331, Phe288, His440, Gln74, Leu282, Trp432, Asn85, and Asp285) were allowed to change their conformation. The visual inspections of each pose of the docked ligands were carried out in order to assure that the binding energies corresponded to the structurally-analogous orientations. The procedure was validated in our previous paper [61].

### 4.9. In Vitro Cytotoxicity Test

The SH-SY5Y human neuroblastoma cell lines (ATCC CRL-2266) were maintained in a1:1 mixture of Eagle’s Minimum Essential Medium and F12 Medium, supplemented with 10% (*v/v*) of foetal bovine serum. The cells were grown at 37 °C in a humidified incubator with 5% CO_2_.

The viability of SH-SY5Y cells was evaluated based on the reduction of the yellow tetrazolium salt (3-(4,5-dimethylthiazol-2-yl)-2,5-diphenyltetrazolium bromide or MTT) to the purple formazan crystals by the metabolically active cells. The cells were seeded onto the 96-well plates at the density of 10,000 cells/well, grown overnight and treated with astragaloside IV at 6, 12.5 or 25 μg/mL. Following 24 h of incubation the medium was replaced with 200 µL of fresh medium containing MTT (final concentration 0.5 mg/mL) and the cells were incubated for a further 4 h at 37 °C. The precipitated formazan was dissolved in 100 µL DMSO and the absorbance of the samples was measured at λ = 540 nm. The mean absorbance measured for the control cells, treated with the solvent (saline), was set to 100% cellular viability and used to calculate the percent of viable cells under each experimental condition.

### 4.10. The Effect of Astragaloside IV on the Zebrafish Embryos

Zebrafish (*Danio rerio*) embryos were obtained from the Experimental Medicine Centre, Medical University of Lublin, Poland. The animals were kept in an incubator under the appropriate environmental conditions (28.5 ± 0.5 °C, light/dark cycle: 14/10 h) up to 96 h post-fertilization (hpf). For the studies, the National Institutes of Health Guide for the Care and Use of Laboratory Animals (Directive 2010/63/EU) was applied. Thus, for the yolk-feeding larvae up to 120 hpf ethical approval is not required. Nevertheless, all attempts were made to minimize the number of animals and their suffering. Immediately after the experiments, the larval zebrafish were euthanized in a15 µM tricaine solution.

In order to assess the effect of astragaloside IV on the developing organism, the zebrafish embryo acute toxicity test was carried out according to the Organization for Economic Cooperation and Development recommendation for the testing of chemicals (Test No. 236) as described previously [62]. One hour after fertilization, viable, perfectly spherical and completely transparent embryos were selected and transferred to 12 well plates (Sarstedt, Germany). The embryos were randomly divided into control and experimental groups. The embryos were kept in each well until the appropriate stage of development was reached of 96 hpf. Two batches of embryos (3 wells per batch, n = 5–6 per well) were kept in 3 mL of zebrafish E3 medium without treatment, i.e., the control group and that was supplemented with 25 µg/mL of astragaloside IV, i.e., the experimental group. Mortality was assessed after 23, 47, 71 and 95 h of exposition. Hatchability was evaluated after 71 and 95 h of exposition. Morphological abnormalities were scored after 95 h of exposition: heartbeat, heart oedema, yolk sac utilization/necrosis, jaw development, eye size, body axis and haemorrhage [62]. Furthermore, to assess muscle function and performance, the touch-evoked response was performed by slight touching of the tail with metal tweezers [63]. The criteria for the larval response were as follows: absent, decreased and normal, as described in detail in our previous paper [64].

### 4.11. Statistical Analysis

The obtained data were pooled together. The Chi-squared test or the Fisher’s exact test was used for statistical purposes.

## 5. Conclusions

The above-described properties of astragaloside IV together with the results of this study confirming its ability to inhibit the action of the acetylcholinesterase enzyme—directly engaged in the process of cognition—place astragaloside IV on the list of compounds that can be drug candidates for the treatment of cognitive impairment diseases. However, it should be noted that the assays for acetylcholinesterase-inhibitory activity were performed by TLC. This type of research needs to be also confirmed in vivo on model organisms. However, in accordance with Etkins’ approach [65] to the determination of biological activity, it is important in the first stage of the experiment to carry out research based on computational as well as biomimetic methods while confirming the safety of living organisms. The docking results are in qualitative agreement with the experimental IC_50_ values, confirming the large potency of astragalosides to be bound into the active site of AChE. It was confirmed that A IV is the most active compound (with the smallest IC_50_ value) among the tested saponins occurring in the roots of *Astragalus mongholicus*. Moreover, no toxic effect was detected in eitherin vitro orin vivo tests. A IV can cross the BBB and has an AChE inhibitory potential comparable to e.g., isoginkgetin from *Selaginella doederleinii Hieron* Selaginellaceae (whole plant), macelignan from *Myristica fragrans Houtt.* Myristicacea, voacangine *Ervatamia hainanensis* Tsiang Apocynaceae (stems), or swatinine-C from *Aconitum* laeve *Ranunculaceae* (tubers) [66].

## Figures and Tables

**Table 1:** Parameters of the Soczewiński-Wachtmeister equation calculated for the tested chromatographic systems.

Chromatographic System	Compound	logkw	s	R^2^
IAM	A I	2.713	6.040	0.992
	A II	2.104	4.715	0.995
	A III	1.807	4.165	0.969
	A IV	1.727	3.965	0.965
CHOL	A I	3.234	5.590	0.985
	A II	3.209	6.570	0.985
	A III	2.627	5.681	0.981
	A IV	2.668	6.000	0.981

**Table 2:** The chemical structures of the tested compounds.

No.	Name	Chemical Structure
1	Astragaloside I	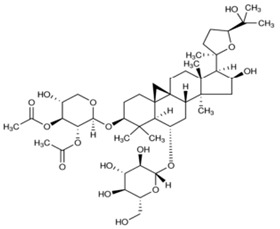
2	Astragaloside II	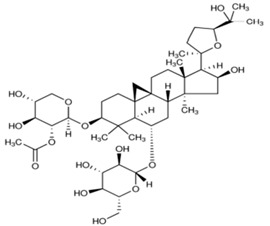
3	Astragaloside III	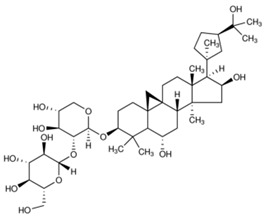
4	Astragaloside IV	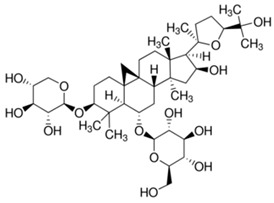

## Data Availability

Data sharing is not applicable to this article.
